# Deep brain stimulation in alpha-synuclein models of Parkinson's disease: Bridging the translational gap

**DOI:** 10.1177/1877718X261459876

**Published:** 2026-06-16

**Authors:** Laura Kondrataviciute, Hanna Weber, Minesh Kapadia, David H Aguirre-Padilla, Mareike Fauser, Taufik A Valiante, William D Hutchison, Luka Milosevic, Alexander Storch, Lorraine V Kalia, Suneil K Kalia

**Affiliations:** 1 Institute of Biomedical Engineering, 127383 University of Toronto, Toronto, Canada; 2 Krembil Research Institute, Toronto Western Hospital, 7989 University Health Network, Toronto, ON, Canada; 3 Department of Neurology, 9187 University of Rostock, Rostock, Germany; 4 Department of Neurology & Neurosurgery, Center Campus, Universidad de Chile, Santiago, Chile; 5 Department of Neurology and Neurosurgery, Medical School, University of Chile, Santiago, Chile; 6 Neuromodulation and Functional Neurosurgery Program, 60765 San Borja-Arriarán Hospital, Santiago, Chile; 7 Department of Biomaterials and Biomedical Technology, 10173 University Medical Center Groningen, University of Groningen, Groningen, The Netherlands; 8 CRANIA, 7989 University Health Network and University of Toronto, Toronto, Canada; 9 KITE, Toronto Rehabilitation Institute, 7989 University Health Network, Toronto, Canada; 10 Division of Neurosurgery Department of Surgery, 7938 University of Toronto, Toronto, Canada; 11 Tanz Centre for Research in Neurodegenerative Diseases, 7938 University of Toronto, Toronto, ON, Canada; 12 Division of Neurology, Department of Medicine, 7938 University of Toronto, Toronto, ON, Canada

**Keywords:** Parkinson's disease, alpha-synuclein, deep brain stimulation, electrophysiology, animal models

## Abstract

Deep brain stimulation (DBS) is an established therapy for advanced medication-resistant Parkinson's disease (PD), yet its ability to alter the course of the disease remains uncertain. Although preclinical research using toxin-induced PD models demonstrate neuroprotective effects, clinical studies in PD patients undergoing DBS have not substantiated these findings. This disconnect may be attributed to factors such as the initiation of DBS late in disease, stimulation protocols targeting symptoms rather than pathology, and the limited translational relevance of animal models lacking hallmark alpha-synuclein (α-Syn) aggregation. Incorporating α-Syn-based models may bridge this gap by facilitating the discovery of early electrophysiological biomarkers of pathological progression, refining stimulation parameters to enhance α-Syn clearance, and assessing if early DBS intervention can mitigate neurodegeneration. Yet, only a limited number of DBS studies have employed α-Syn models to date. This review examines the translational gap between preclinical neuroprotection claims and clinical outcomes, focusing on how α-Syn-based models could resolve current limitations in DBS research. Prioritizing these models could clarify whether DBS has the potential to extend beyond symptomatic relief and directly engage PD's underlying neurodegenerative mechanisms. Achieving this goal requires systematic investigation of DBS influences on α-Syn accumulation and its electrophysiological correlates in disease-relevant models.

**Plain language summary title:**

Deep Brain Stimulation in Alpha-Synuclein Models of Parkinson's Disease: Bridging the Translational Gap

## Plain language summary

Deep brain stimulation (DBS) is an effective treatment that helps people with Parkinson's disease manage their movement symptoms, like tremors and stiffness. But while it provides relief, a big question remains: could DBS also slow down the disease progression itself? Studies in animals suggest it might protect brain cells, but these promising results have not yet translated to human patients. The reason may lie in key differences between research and real-world treatment.

Most animal studies use methods that do not fully replicate Parkinson's disease in humans—particularly the gradual buildup of harmful alpha-synuclein protein aggregates that are linked to Parkinson's disease. Additionally, DBS is typically given to patients only after their symptoms become severe, when significant damage has already occurred. Current DBS settings are also optimized for symptom control rather than targeting the disease process directly.

Research using alpha-synuclein-based animal models which may better mimic human disease hints that DBS might have untapped potential. Some studies show it could help clear alpha-synuclein aggregates or protect brain cells, while others find no such benefit. This mixed evidence tells us we need a deeper understanding of how timing, brain targets, and stimulation settings influence DBS's effects.

Looking ahead, researchers are exploring whether DBS could be used earlier—perhaps even before symptoms appear—to intervene in the disease process. The goal is to shift DBS from solely managing symptoms to potentially slowing or even preventing disease. While much work remains, these advances could one day transform how we treat Parkinson's disease, offering hope for more than just symptom relief.

## Introduction

Parkinson's disease (PD) is the fastest-growing neurodegenerative movement disorder, characterized by the progressive degeneration of dopaminergic neurons in the *substantia nigra pars compacta* (SNpc) and the pathological accumulation of alpha-synuclein (α-Syn) aggregates.^1^ Although no existing treatment alters the underlying neurodegenerative process, levodopa remains the first-line pharmacological therapy for symptomatic management, despite the emergence of long-term complications such as motor fluctuations and dyskinesias.^2–4^ In patients with advanced disease who exhibit poor responsiveness to medication or severe side-effects, deep brain stimulation (DBS) targeting the subthalamic nucleus (STN) or globus pallidus internus (GPi) has emerged as a highly effective neurosurgical intervention. By delivering continuous high-frequency electrical pulses, DBS modulates aberrant basal ganglia network activity and improves Parkinsonian motor symptoms.^5^

**Figure 1.**
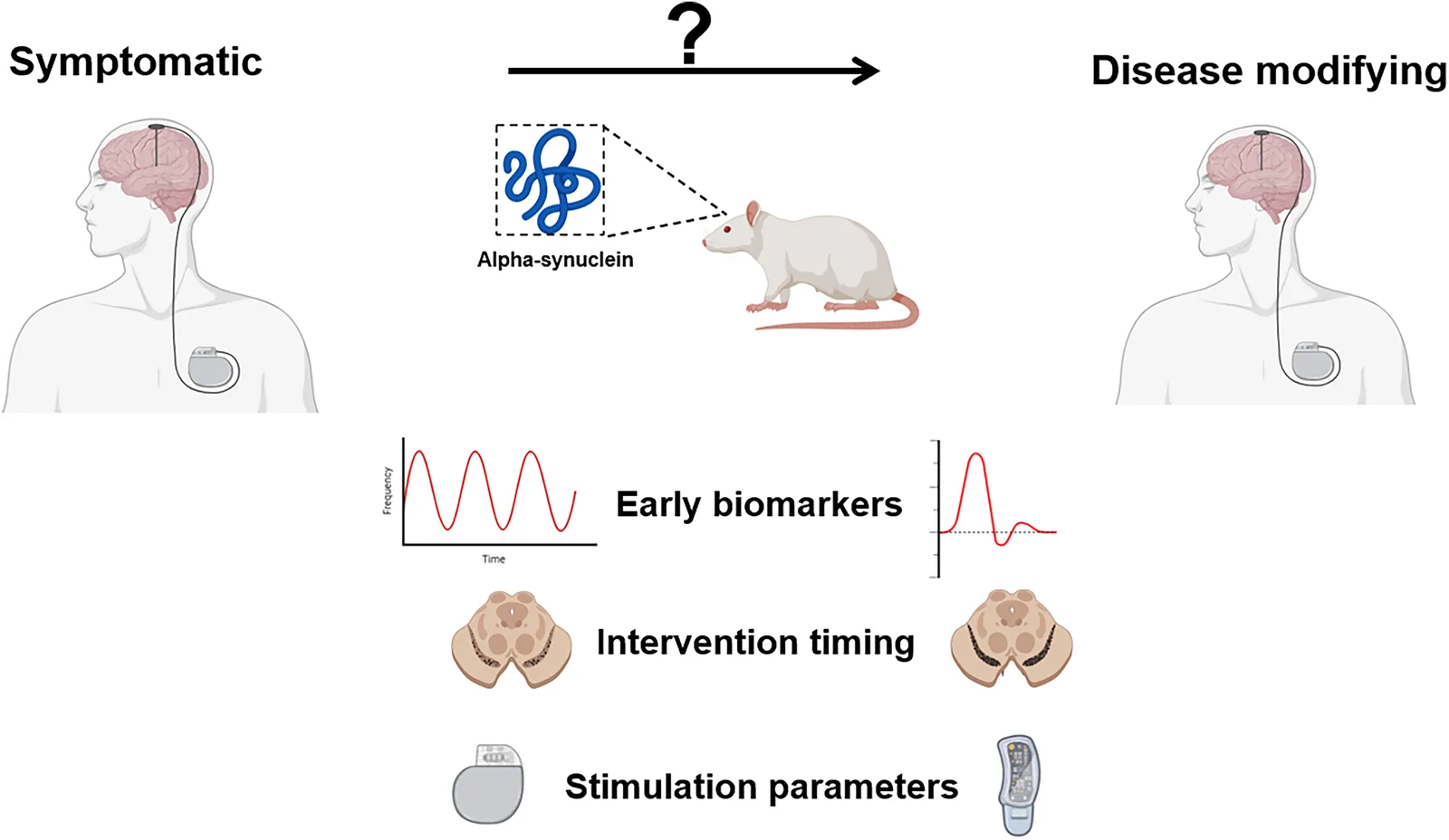
Visual abstract.

DBS has been shown to exert effects across multiple levels of neural organization, including modulation of local neuronal firing patterns, reshaping of network-wide oscillatory activity, and induction of neurochemical alterations.^6,7^ This multifaceted mode of action raises the possibility that DBS can engage disease-modifying processes in PD, beyond its established role in symptomatic relief. Supporting this notion, preclinical studies using toxin-based models have reported neuroprotective outcomes such as preservation of nigrostriatal dopaminergic neurons and upregulation of neurotrophic factor expression.^8–14^ Despite these encouraging findings, clinical evidence has not yet validated a disease-modifying role for DBS. This translational discrepancy may be attributable to three key factors: (1) the predominant use of preclinical models that lack PD's hallmark α-Syn pathology and progressive nature, (2) the application of DBS primarily at advanced disease stages, and (3) the use of stimulation protocols optimized solely for motor symptom control. Here, we focus on α-Syn-based models precisely because they can mimic key elements of human PD, and, therefore, enable clinically relevant investigations of DBS's disease-modifying potential. (Figure 1)

## Timing and targets: the neuroprotection paradox in PD DBS research

The majority of preclinical DBS studies have relied on toxin-based models, notably 6-hydroxydopamine (6-OHDA) in rodents and 1-methyl-4-phenyl-1,2,3,6-tetrahydropyridine (MPTP) in non-human primates, which trigger rapid and targeted degeneration of dopaminergic neurons.^15,16^ These models have been extensively reviewed in rodent^17,18^ and non-human primate systems,^19^ but both species have shown neuroprotective effects of DBS in the STN. In MPTP-treated monkeys, STN-DBS and STN lesioning yielded comparable dopaminergic neuron preservation in the SNpc, an effect attributed to reduced glutamate-mediated excitotoxicity via decrease of the STN input to the SNpc.^20^ In MPTP-treated rats and non-human primates STN-DBS preserved dopaminergic cells in the SNpc by increasing mitophagy (via mTOR suppression) and reducing oxidative stress via upregulation of the superoxide dismutase enzyme and glutathione.^21^ In 6-OHDA-lesioned rats, neuroprotective effects of DBS have been linked to neurogenesis,^22^ enhanced brain-derived neurotrophic factor tropomyosin receptor kinase B (BDNF/TrkB) signaling,^23^ and modulation of striatal genes regulating growth, apoptosis, and synaptic transmission.^24^ However, these acute models produce dopaminergic neuron death through less relevant to PD mechanisms, replicating a dopamine-deficient phenotype without progressive α-synucleinopathy, which likely explains their poor translational record for disease modification.^25^

Long-term clinical follow-up of patients undergoing STN-DBS has not provided evidence for disease modification, particularly in the absence of rigorously blinded trial designs.^26,27^ Postmortem analyses of long-term STN-DBS recipients have revealed neither preservation of SNpc dopaminergic neurons nor reductions in α-Syn burden,^28,29^ and neuroimaging studies showed similar rates of dopaminergic decline in DBS-treated and untreated patients.^30^ The rapid symptom recurrence following stimulation withdrawal further argues against a true neuroprotective effect.^31^ Instead, most observed benefits of DBS appear to derive from symptomatic improvements—enhanced quality of life rather than slowed disease progression—and these effects tend to diminish over time, which is inconsistent with true pathological reversal.^26,32^ A recent pilot randomized clinical trial suggested that early STN-DBS application reduced motor complications over an 11-year follow-up compared to pharmacotherapy alone.^33^ However, such improved long-term outcomes do not necessarily equate to disease modification at the molecular level. These benefits may instead reflect activity-dependent stabilization of maladaptive network dynamics, delaying functional symptom manifestation without directly altering disease pathophysiology.

Nevertheless, the absence of detectable disease modification in unselected patient populations does not preclude the possibility that specific subgroups might derive such benefits. Therapeutic outcomes vary substantially across subtypes of PD^34–36^ —a consideration with direct implications for evaluating DBS as a disease-modifying intervention.^34–36^ For example, one multi-cohort analysis found that glucocerebrosidase (GBA) mutation carriers who underwent STN-DBS exhibited accelerated cognitive decline compared to non-carriers.^37^ However, a large Japanese cohort study reported no significant differences in cognitive or motor trajectories between GBA carriers and non-carriers over five years of follow-up, although GBA carriers exhibited worsened motor symptoms in the off-medication/off-stimulation state at the five-year time point.^38^ These conflicting findings underscore that PD is biologically heterogeneous and that any disease-modifying effects of DBS—if they exist—would likely depend critically on genetic background, disease stage, and individual network pathophysiology.

This clinical heterogeneity as well as the fundamental differences in disease pathophysiology and timing of intervention may help explain the observed disconnect between neuroprotective outcomes reported in preclinical DBS studies and the lack of disease-modifying effects in clinical populations. Toxin-based models lack progressive α-Syn pathology, which drives circuit dysfunction (through synaptic impairment and network hypersynchrony),^39^ as well as progressive neurodegeneration through prion-like spreading and proteostatic failure.^40^ Without these α-Syn-dependent circuit disruptions, DBS modulates artificially simplified networks, limiting translational relevance of toxin-based models to clinical PD. Moreover, the acute and severe dopamine depletion induced by toxins generates immediate circuit-level disruptions that are physiologically distinct from the gradual α-Syn induced neurodegeneration seen in PD – a difference clearly demonstrated by comparing behavioral outcomes in acute versus gradually depleted 6-OHDA mouse models.^41^ Mechanisms proposed to underlie neuroprotection in preclinical settings (e.g., reduced glutamatergic excitotoxicity, upregulation of BDNF) may not effectively engage the same chronically remodeled basal ganglia-thalamocortical networks characteristic of late-stage PD. Lastly, while preclinical studies often initiate DBS concurrently with or shortly after the neurotoxic insult, clinical intervention with DBS is typically implemented years, if not decades, after pathological onset.^42^ This temporal disconnect raises the critical question of whether earlier application of DBS prior to extensive network remodeling and neuronal loss might unlock its disease-modifying potential.

## Repurposing DBS for molecular intervention

Emerging evidence indicates that electrical stimulation may exert direct effects on α-Syn pathology.^43,44^ *In vitro* experiments have shown that high frequency stimulation, mimicking DBS parameters used for PD, can reduce intracellular levels of α-Syn oligomers in rat's cortical neurons.^44^ This reduction in α-Syn levels appears to be associated with a rescue in α-Syn-mediated autophagic impairment.^43^ Electrical stimulation has been reported to enhance autophagic flux,^45^ which may facilitate the clearance of aggregated α-Syn and potentially interfere with mechanisms involved in its trans-synaptic propagation.^46^

Beyond autophagy-mediated clearance, α-Syn propagation is increasingly recognized as an activity-dependent process. α-Syn has been shown to be secreted in a calcium-dependent manner via exosomal pathways, indicating that neuronal activity and intracellular calcium dynamics regulate its extracellular release.^47^ Experimental modulation of exocytosis significantly alters α-Syn secretion and propagation dynamics, supporting the concept that vesicular release pathways are critical determinants of intercellular transmission.^48^ In parallel, pharmacological suppression of neuronal activity has been reported to attenuate α-Syn propagation, whereas enhanced neuronal excitability can facilitate its spread within connected circuits.^49^ In this context, DBS-induced modulation of neuronal firing patterns and network synchrony may critically shape α-Syn dynamics at the level of release, uptake, and interregional transmission. Rather than acting solely through intracellular degradation pathways, DBS may therefore influence the balance between secretion, propagation, and clearance of α-Syn, providing a mechanistic framework that links circuit-level intervention to molecular pathology.

Despite these promising findings, their clinical applicability is likely to be constrained by the timing of DBS intervention. Currently, DBS is reserved for patients with advanced disease, typically initiated more than 7 years after the onset of motor symptoms and following at least 1.5 years of motor complications.^50,51^ By this late stage, substantial dopaminergic neuron loss has already occurred, making functional recovery unlikely even if α-Syn clearance were achieved.

This highlights the distinctive utility of α-Syn rodent models, which can offer researchers controlled platforms to: (1) delineate the optimal therapeutic window for α-Syn-directed DBS by varying the timing of intervention across disease stages, (2) refine stimulation parameters to theoretically maximize α-Syn clearance, (3) identify early electrophysiological or molecular biomarkers that could inform DBS timing decisions in a clinical setting; and (4) assess whether pre-symptomatic intervention can attenuate or halt pathological progression. Collectively, such investigations have the potential to reposition DBS from a symptomatic treatment to a disease-targeted approach addressing PD's underlying molecular underpinnings.

## Mixed and limited findings on neuroprotective effects of DBS in α-synuclein animal models

A PubMed search using the terms ((rat) OR (mice)) AND ((DBS) OR (deep brain stimulation)) AND (6-OHDA) NOT (review) yields 158 results, whereas substituting (6-OHDA) with (alpha-synuclein) returns only 16 entries - indicating a substantial bias in the preclinical DBS literature toward toxin-based paradigms.

α-Syn-based models have been leveraged in only a limited number of DBS studies to date.^52,53^ In one such study, chronic high-frequency (130 Hz) STN-DBS administered for three weeks in rats overexpressing mutant A53 T α-Syn via adeno-associated virus (AAV1/2-A53 T) resulted in a ∼29% increase in tyrosine hydroxylase-positive (TH^+^) cells in the SNpc compared to stimulation-off controls.^52^ This was accompanied by a near-complete restoration of motor performance in the single-pellet reaching task, comparable to rats injected with empty vector (EV) virus control.^52^ Interestingly, functional gains following STN-DBS were not accompanied by an alteration in homovanillic acid/dopamine *(*HVA/DA) ratio, leaving the underlying neuroprotective mechanism unresolved.^52^ In follow-up work using the same AAV1/2-A53 T model, the authors demonstrated that three weeks of STN-DBS at 130 Hz reduced the formation of pathological TrkB receptor clusters, which are implicated in impaired synaptic plasticity and dopaminergic denervation in PD.^53^ This reduction in TrkB cluster formation was associated with partial restoration of dopaminergic innervation in the striatum, suggesting DBS-mediated rescue of synaptic and neuroplastic processes.^53^ Effects of DBS across α-Syn-based PD rodent models are summarized in Table 1.

**Table 1.** Effects of deep brain stimulation (DBS) across α-synuclein (α-Syn)-based Parkinson's disease (PD) rodent models.

Model	Strain, weight, sex	Stimulation protocol	Neuro-degeneration in SN	α-Syn accumulation in SN	Behavior	Reference
AAV2/5 WT α-Syn overexpression in rats	Sprague-Dawley; ≈250 g; Male	Unilateral 130 Hz STN, PW: 60 μs, Amp: 30–50 μA; Dur: 26 d.	↔ DA	↔	Worsened motor skills (↑ Forelimb asymmetry)	^54^
AAV-V1S/-SV2 WT α-Syn overexpression in rats	Sprague-Dawley; 250–280 g; Female	Unilateral 130 Hz SN or STN, PW: 60 μs, Amp: 100 μA; Dur: 1 h.	↔ DA	**↓** SN-DBS **↔** STN-DBS	NS	^44^
PFF mice	Fischer 344; NS weight; Male	Unilateral 130 Hz STN, PW: 60 µs, Amp: ∼50 µA; Dur: 30 d.	↔ DA	NS	NS	^55^
AAV1/2 A53 T α-Syn in rats	Sprague-Dawley; 280 g; Male	Unilateral 130 Hz STN, PW: 60 μs, Amp: 20% below side effects. Dur: 21 d.	↓ DA	NS	Improved motor skills (↑ Relative success in single pellet reaching task)	^52^

↑: increased; ↔: no significant change; ↓: decreased; NS: not studied/stated; AAV: adeno-associated virus; Amp: amplitude; DA: nigral dopaminergic neurons; DBS: deep brain stimulation; Dur: duration; GPe: globus pallidus externus; MCx: motor cortex; PFF: post- α-Syn-preformed fibrils; PW: pulse width; SN: substantia nigra; STN: subthalamic nucleus; WT: wild-type.

In contrast to these findings, no neuroprotective benefits were observed using unilateral AAV2/5-mediated overexpression of wild-type human α-Syn in rats.^54^ In this study, 28 days of continuous 130 Hz STN-DBS failed to preserve SNpc dopaminergic neurons or improve motor performance.^54^ Moreover, STN-DBS exacerbated forelimb akinesia in this model, potentially due to asymmetric dopaminergic signaling driven by bilateral striatal dopamine release during stimulation.^54^ Similarly, in the α-Syn-preformed-fibril (PFF) rat model, 30 days of STN-DBS did not reduce α-Syn inclusion load, microgliosis, or astrogliosis in the SNpc.^55^ Nevertheless, the same study reported elevated BDNF levels in the striatum and partial re-establishment of corticostriatal BDNF signaling, suggesting that DBS may still engage trophic support pathways even in the presence of established α-Syn pathology.^55^ Importantly, Lee and colleagues^44^ demonstrated that while STN stimulation showed no effect on α-Syn levels, SNpc stimulation significantly reduced both α-Syn oligomers and total α-Syn levels in AAV vector-based rat model, highlighting how target selection may critically determine DBS's capacity to modify α-Syn pathology.

## Electrophysiological insights: A critical gap in α-synuclein models

The development of effective neuroprotective DBS strategies hinges on addressing two core challenges: (1) determining the optimal timing for intervention, and (2) sustaining effective stimulation parameters as disease pathology evolves. Central to the first is the identification of reliable electrophysiological biomarkers that reflect α-Syn pathological progression, such as disease-specific oscillatory activity or altered network dynamics that emerge during prodromal stages and correlate with molecular pathology. These biomarkers could help to define a critical therapeutic window during which DBS may prevent further α-Syn aggregation or propagation and avert irreversible neurodegeneration. However, even with precise temporal targeting, static stimulation protocols may prove insufficient, as DBS-mediated α-Syn clearance is likely contingent upon underlying network states.^8^ This highlights the need for closed-loop stimulation systems capable of dynamically adjusting DBS output in response to real-time electrophysiological feedback and where feasible, molecular indicators of α-Syn burden.^56^ By adaptively adjusting stimulation, intensifying during phases of active protein aggregation and tapering as pathology subsides, such systems could theoretically sustain therapeutic efficacy and mitigate the diminishing effects observed in long-term DBS patients.^26^ Although α-Syn rodent models provide an essential framework to explore these dynamics, their broader applicability will depend on delineating the spatiotemporal evolution of electrophysiological signatures in parallel with α-Syn pathology.

Establishing correlations between electrophysiological signals and α-Syn pathology necessitates comprehensive phenotypic characterization of α-Syn-based rodent models. Andersen *et al*. investigated single unit activity in the STN under urethane anesthesia in a unilateral AAV2/5-mediated human wild-type α-Syn overexpression rat model, focusing on the modulatory role of leucine-rich repeat kinase 2 (LRRK2).^57,58^ This model exhibited PD-like STN burst firing without alterations in firing rate compared to green fluorescent protein (GFP) or EV injected controls, despite retaining ∼80% of ipsilateral striatal TH expression relative to the contralateral hemisphere.^57^ Notably, acute inhibition and genetic knockout of LRRK2 both attenuated burst firing, with knockout increasing firing rate suggesting that STN hyperexcitability may be partially LRRK2-dependent but not solely driven by synucleinopathy.^57^ Interestingly, the same group later reported that chronic LRRK2 inhibition with PFE-360 fails to normalize aberrant STN activity.^58^ These findings carry clinical relevance, as LRRK2 mutation carriers often present with Parkinsonism in the absence of classical Lewy body pathology,^59^ implying that individualized DBS paradigms may be required for genetically distinct PD subtypes.

Electrophysiological investigations in α-Syn models beyond the STN have largely focused on other brain regions or employed local field potential (LFP) recordings rather than single-unit analyses. For example, in an AAV6-mediated human wild-type α-Syn overexpression rat model with ∼48% nigral TH + cell loss, Brys *et al*. observed no alterations in the firing rates of neurons in the motor cortex or dorsolateral striatum relative to EV-injected controls.^60^ Findings on beta-band LFP activity, an established signature of PD,^61^ are inconsistent across α-Syn models. Elevated cortical beta power was reported 12 weeks after dorsal striatum and motor cortex PFF seeding in mice, despite the absence of overt motor deficits,^62^ whereas no difference in beta power was detected in the same regions in the aforementioned AAV6-mediated human wild-type α-Syn overexpression rat model.^60^ Although in different α-Syn models, these discrepancies suggest that beta-band oscillations may not directly mirror motor impairment. Supporting this, Hofman *et al*. recently demonstrated that high beta (20–35 Hz) power in the STN, but not in the motor cortex, correlated with both motor deficits and dopaminergic cell loss in the AAV1/2-A53 T rat model of PD,^63^ highlighting the region- and circuit-specific electrophysiological consequences of synucleinopathy. Summary of electrophysiology findings in α-Syn-based PD rodent models is provided in Table 2.

**Table 2.** Summary of electrophysiology findings in α-synuclein (α-Syn)-based Parkinson's disease (PD) rodent models.

Model	Neurodegeneration	Target	Findings	Reference
PFF mice	↔ NeuN cell loss across MCx 12 weeks post seeding vs monomer	Primary MCx in forced motion	↑ Beta power in MCx 12 weeks after PFF seeding vs monomer controls. Progressive ↑ firing rate in MCx of Str- and MCx-seeded animals vs monomer-seeded controls.	^62^
AAV2/5 human WT α-Syn overexpression in rats	∼20% reduction in Str TH + cells vs contralateral side	STN under urethane anesthesia	↑ Bursty firing pattern vs GFP overexpression vs EV group. ↑ CV vs GFP overexpression vs EV group. ↔ Firing rate vs EV group.	^57,58^
AAV6 human WT α-Syn overexpression in rats	∼ 48.5% reduction in SN TH + cells vs contralateral side	Str and MCx in awake state	↔ Firing rate between hemispheres in Str, MCx. No beta oscillations in Str, MCx.	^60^
AAV1/2 A53 T α-Syn overexpression in rats	∼ 24% (high conc.) & ∼13% (low conc.) reduction in SN TH + cells vs EV control group	STN and MCx in awake state	Progressive ↑ beta oscillations in MCx and STN. High beta activity in STN but not MCx correlated with motor impairment and dopaminergic neurodegeneration.	^63^

Abbreviations: ↑: increased; ↔: no significant change; ↓: decreased; AAV: adeno-associated viral vector; conc.: concentration; CV: coefficient of variation of interspike interval; EV: empty vector; GFP: green fluorescent protein; MCx: motor cortex; NeuN: neuronal nuclei positive cells; PFF: post- α-Syn-preformed fibrils; SN: substantia nigra pars compacta; STN: subthalamic nucleus; Str: striatum; TH: tyrosine hydroxylase; WT: wild-type.

The paucity of electrophysiological studies in α-Syn models underscores a critical gap in the field. Divergent findings may stem from numerous experimental variables, including differences in the model (e.g., age, sex, viral serotype and titer, α-Syn variant, injection coordinates, extent of neurodegeneration), recording methodologies (e.g., anesthetized vs awake, restrained vs freely moving) and inconsistent definitions of beta activity (e.g., burst analysis vs average power). Without systematic mapping of how electrophysiological alterations evolve alongside molecular and cellular pathology, the potential to use α-Syn models to uncover circuit-level biomarkers and mechanisms amenable to DBS remains largely untapped.

## Future directions of DBS research

As the therapeutic landscape of PD evolves, DBS may be repositioned from a purely symptomatic therapy toward an integral component of multimodal disease-modifying strategies. Several specific priorities emerge from this review. First, systematic parameter optimization in α-Syn rodent models is needed to identify stimulation parameters (frequency, amplitude, target) that maximize α-Syn clearance and autophagic flux, which may differ from parameters optimized for motor symptom control. Second, biomarker-guided patient stratification—integrating genetic status, electrophysiological signatures, and emerging α-Syn PET or fluid biomarkers—will be essential for identifying prodromal or early-stage patients most likely to benefit and for monitoring target engagement. Third, adaptive closed-loop stimulation systems, informed by longitudinal electrophysiological recordings in α-Syn models, could enable dynamic adjustment of stimulation in response to disease-state biomarkers, potentially outperforming static open-loop paradigms. Fourth, combinatorial approaches integrating DBS with molecular therapies (e.g., anti-α-Syn antibodies, aggregation inhibitors, autophagy enhancers, or gene therapies targeting GBA or LRRK2) warrant preclinical investigation for synergistic effects. Fifth and most fundamentally, α-Syn models offer the unique opportunity to define the therapeutic window for disease-modifying DBS by systematically varying intervention timing from pre-symptomatic through late-stage pathology. Collectively, these directions highlight the potential repositioning of DBS from a purely symptomatic therapy toward a precision tool against PD's molecular drivers.

## Conclusion

While DBS remains a treatment for PD motor symptoms management, its potential to modify disease progression remains limited by two critical gaps: the predominant use of toxin-based models that fail to replicate progressive α-synucleinopathy, and clinical intervention that typically occurs too late in the disease course. Emerging evidence from α-Syn models, though still limited in number, suggests DBS may directly engage pathological mechanisms, including autophagy-mediated α-Syn clearance and oligomer reduction with potential neuroprotective effect. These findings highlight the untapped potential of α-Syn models to resolve key translational questions: (1) How can early electrophysiological biomarkers guide intervention before irreversible neuronal loss? (2) Can stimulation parameters be optimized for pathology clearance rather than symptom control? And crucially, (3) Could pre-symptomatic DBS, timed to the initial stages of α-Syn spread, prevent neurodegeneration altogether? PD DBS research with α-Syn-based models may enable a paradigm shift—from viewing DBS solely as symptomatic therapy to leveraging its circuit-modulating power as a precision tool against PD's molecular drivers.
